# A potential link between aberrant expression of ECRG4 and atrial fibrillation

**DOI:** 10.3389/fonc.2023.1031128

**Published:** 2023-02-22

**Authors:** Zuojing Zhang, Wei Wang, Yuxin Zhang, Xingji You, Jingxiang Wu

**Affiliations:** ^1^ Department of Anesthesiology, Shanghai Chest Hospital, Shanghai Jiao Tong University, School of Medicine, Shanghai, China; ^2^ School of Medicine, Shanghai University, Shanghai, China

**Keywords:** esophageal cancer-related gene-4, myocardial injury, tumor suppressor gene, atrial fibrillation, radical surgery for esophageal cancer

## Abstract

Esophageal cancer-related gene-4 (ECRG4), a 148-amino acid propertied and new tumor suppressor, is initially cloned from the normal esophageal epithelium. ECRG4 was found to be expressed not only in esophageal tissues but also in cardiomyocytes. Previous studies demonstrated that ECRG4 is constitutively expressed in esophageal epithelial cells, and its degree of downregulation is directly proportional to prognosis in patients with esophageal cancer. In the heart, ECRG4 shows greater expression in the atria than in the ventricles, which accounts for its heterogeneity. Downregulation of ECRG4 expression level correlates with esophageal cancer, as well as myocardial injuries and arrhythmias. As a result, this review summarizes the possible susceptibility gene, ECRG4 and its associated molecular mechanisms in cancer patients with atrial fibrillation and myocardial injury. The review begins by describing ECRG4’s biological background, discusses its expression in the cardiovascular system, lists the clinical and animal research related to the downregulation of ECRG4 in atrial fibrillation, and focuses on its potential role in atrial fibrillation. Downregulation of ECRG4 may increase the risk of atrial fibrillation by affecting ion channels, MMPs expression and inflammatory response. We will then discuss how ECRG4 can be used in the treatment of tumors and arrhythmias, and provide a novel possible strategy to reduce the occurrence of perioperative cardiovascular adverse events in patients with tumors such as esophageal cancer and gastric cancer.

## Introduction

Esophageal cancer-related gene-4 (ECRG4) is a newly identified tumor suppressor gene and a sentinel molecule for maintaining tissue homeostasis. Recent research has revealed that ECRG4 expression is quite distinct and is present in esophageal squamous epithelial cells as well as the sinoatrial node, atrioventricular node, atrial and ventricular cells. Additional investigations further concluded that ECRG4 could maintain cardiac homeostasis and regulate cardiac rhythm while it downregulation may contribute to atrial fibrillation (AF) (1). Moreover, ECRG4 is most likely a hypoxic sensor and may be related to myocardial ischemia (1). Notably, ECRG4 is also a tumor suppressor gene that can prevent esophageal cancer cell proliferation (2). Downregulation of this gene expression increases the risk of esophageal cancer and is strongly linked to a poor patient prognosis (2–4). Epidemiological studies also suggest a strong correlation between AF and various tumors. For instance, the risk of colorectal cancer in AF patients was ten times greater than in those without AF, according to a case-control study (5). Furthermore, reduced ECRG4 expression in esophageal cancer patients is associated with an increased incidence of myocardial injury and atrial fibrillation (2, 6). Collectively, the above studies show that ECRG4 may be implicated in both tumorigenesis and cardiovascular adverse events. Herein, we describe the ECRG4’s biological background, discuss its expression in the cardiovascular system, list the clinical and animal research related to the downregulation of ECRG4 in atrial fibrillation, and focus on its potential role in atrial fibrillation. We will then discuss how ECRG4 can be used in the treatment of tumors and arrhythmias, and provide a novel possible strategy to reduce the occurrence of perioperative cardiovascular adverse events in patients with tumors such as esophageal cancer and gastric cancer.

## The biological background of ECRG4

**Figure 1:**
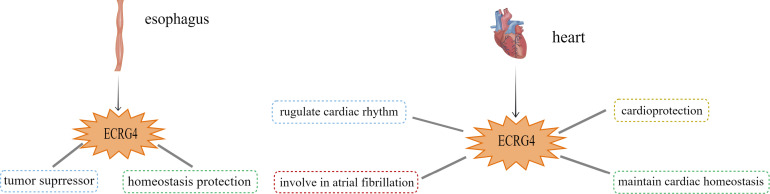
Potential functions of ECRG4 in the esophagus and cardiovascular system.

Su et al. first discovered ECRG4 in normal human esophageal epithelial cells (7), and it was eventually localized in the c2orf40 locus of chromosome 2, which consists of four exons spanning approximately 14.9 kilobases (4, 8). The initial bioinformatic analysis and subsequent biochemical characterization indicate that the protein encoded by ECRG4 is a hormone-like secretory protein. The ECRG4 gene also encodes a protein with a molecular weight of 17KDA, and peptides with different molecular weights associated with the ECRG4 protein were also identified. Most of the tumor suppressor genes are usually intracellular or membrane proteins. Unlike most tumor suppressor genes, which are usually membrane or intracellular proteins, ECRG4 is a 148 amino acid propeptide that is covalently linked to the amino end on cell surfaces (9, 10). Since ECRG4 is attached to the cell surface, it acts as a “sentinel” in maintaining tissue homeostasis (**Figure 1**). The presence of ECRG4 on the cell surface suggests that its homeostasis is maintained. After tissue injury, ECRG4 can quickly detach from the cell surface (within 24 hours), thereby increasing tissue injury responses like vascular leakage, immune cell infiltration, and cell proliferation (in 2-4 days). The injury response gradually disappears during the healing process as ECRG4 returns to the cellular surface (usually in 6-7 days) (11–13).

Previous studies demonstrate that ECRG4 is constitutively expressed in esophageal epithelial cells, and its degree of downregulation is directly proportional to prognosis in esophageal cancer patients (**Figure 1**) (2). Studies have shown that ECRG4 may induce the downregulation of COX-2 through the NF-КB pathway, thereby inhibiting tumor growth in esophageal squamous cell carcinoma (ESCC) (2). Other Studies have found that ECRG4 can directly interact with ECRG1 to up-regulate the expression of p21, induce G1 phase arrest of cell cycle, and inhibit the proliferation of cancer cells (3). Further research revealed that ECRG4 was down-regulated to varying degrees in gastric cancer (14, 15), breast cancer (16, 17), hepatocellular cancer (18, 19), nasopharyngeal cancer (20–22), laryngeal cancer (23), bladder cancer (24, 25), glioma (26), colorectal cancer and prostate cancer (26–28). These findings suggest that ECRG4 plays a tumor-suppressive role. Various other cells/tissues, such as the adrenal gland, choroid plexus, cardiomyocytes, and conduction system, also express ECRG4. ECRG4 is known to regulate inflammation (11–13), induce neuronal senescence (29), participate in the formation of atrial fibrillation (30), and possibly act as a hypoxia sensor, contributing to myocardial injury.

## ECRG4: Expression in the cardiovascular system

In 2017 Mirabeau et al. uncovered ECRG4 as a new secretory peptide in mouse endocrine tissues as well as in other locations, including the pituitary, adrenal gland, pancreas, choroid plexus, and the atrioventricular node of the heart (8). Notably, ECRG4 mRNAs are expressed in heart (31). It was found that ECRG4 was expressed in the sinoatrial node, atrium and ventricle, and ECRG4 expression was higher in atrium (30, 31). Porzionato et al. used immunohistochemical analysis to show that ECRG4 was uniformly expressed in normal rat atrial myocytes while only expressed in sporadic ventricular myocytes (31). Professor Dang further discovered that down-regulating ECRG4 in atrial myocytes activated pro-inflammatory cascade and genes involved in heart remodeling, which participated in the occurrence of atrial fibrillation, and concluded that the normal expression of ECRG4 could maintain cardiac homeostasis (**Figure 1**) (30). Other studies have shown that ECRG4 promotes cardiovascular homeostasis and prevents atrial fibrillation by regulating the response to ischemia/hypoxia (32).

## ECRG4 downregulation is associated with atrial fibrillation: Clinical and animal research

**Figure 2:**
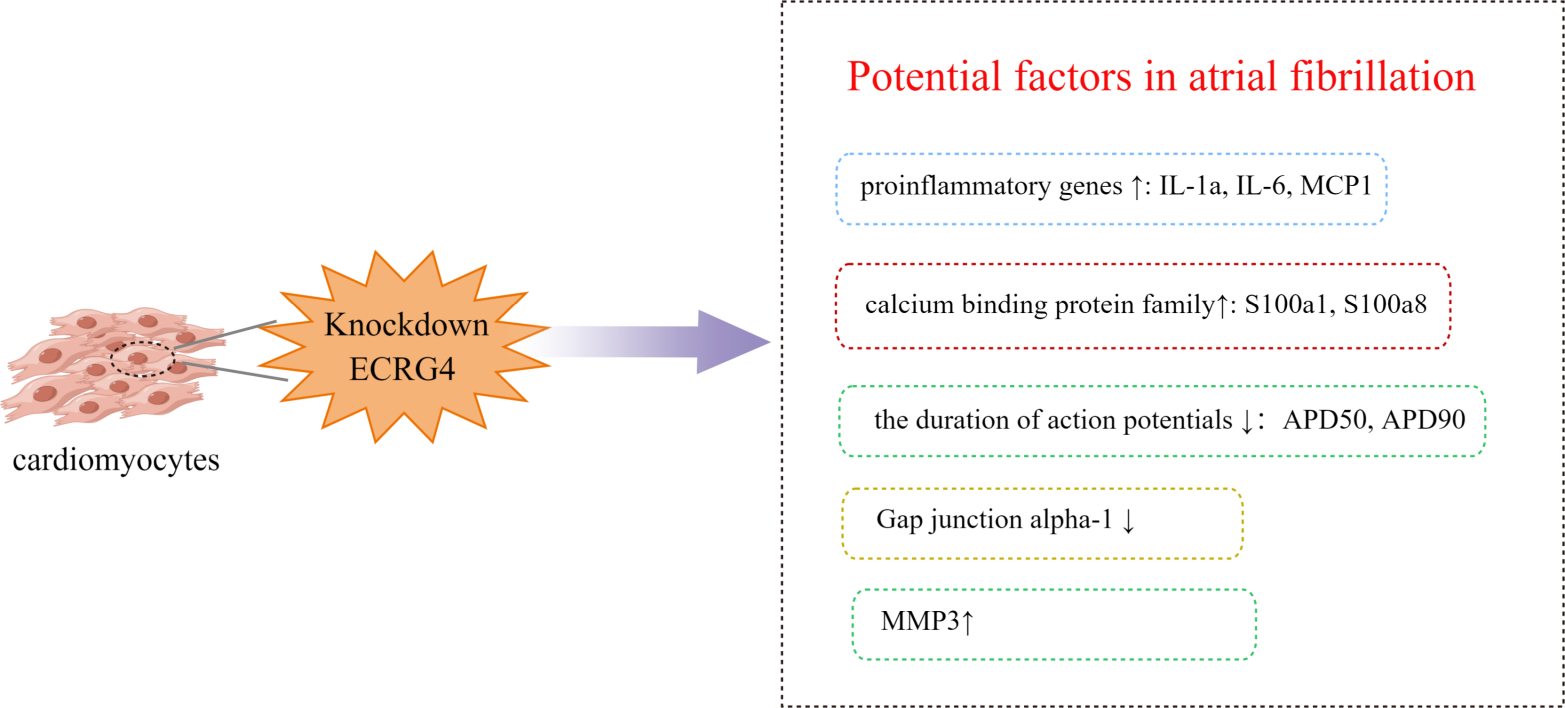
Mechanism of atrial fibrillation induced by down-regulation ECRG4.

Atrial fibrillation is the most common sustained cardiac tachyarrhythmia encountered by physicians, with an ever rising incidence globally. Studies have found that ECRG4 is implicated in the pathogenesis of atrial fibrillation (**Figure 2**). Moreover, since ECRG4 is a tumor suppressor gene, the downregulation of ECRG4 may also be a risk factor for atrial fibrillation in cancer patients. Extensive epidemiological data show a strong correlation between AF incidence and cancer, which has garnered widespread attention. Erichsen found a tenfold increase in the incidence of colorectal cancer in patients with atrial fibrillation (5). A cohort study found that the cancer diagnosis rate in newly diagnosed AF patients increased 5-fold compared to the expected cancer incidence in the general population after a 3-month follow-up (33). Consistently, Conen found that cancer patients experienced twice the incidence of AF during surgery as non-tumor patients (34). Another clinical control study showed that in esophageal cancer patients with intraoperative hypothermia, the incidence of postoperative myocardial injury could rise to 31.4%, while the incidence of atrial fibrillation (AF) was 14.3% (6), significantly higher than the myocardial injury incidence of 8% in other non-cardiac surgeries (35) and 2.9% incidence of atrial fibrillation in thoracoscopic lung cancer surgery (36). Previous studies reported that the incidence of new-onset atrial fibrillation after radical esophagectomy was 12-37% (37–47). Atrial fibrillation may also be linked to lower ECRG4 expression in other patients besides tumor patients Five suitable atrial appendage specimens from patients with rheumatic heart disease with or without atrial fibrillation were further collected clinically to investigate the expression of ECRG4 in the heart, and immunohistochemistry confirmed the downregulation of ECRG4 in the atrial appendage of patients with atrial fibrillation (30).

**Figure 3:**
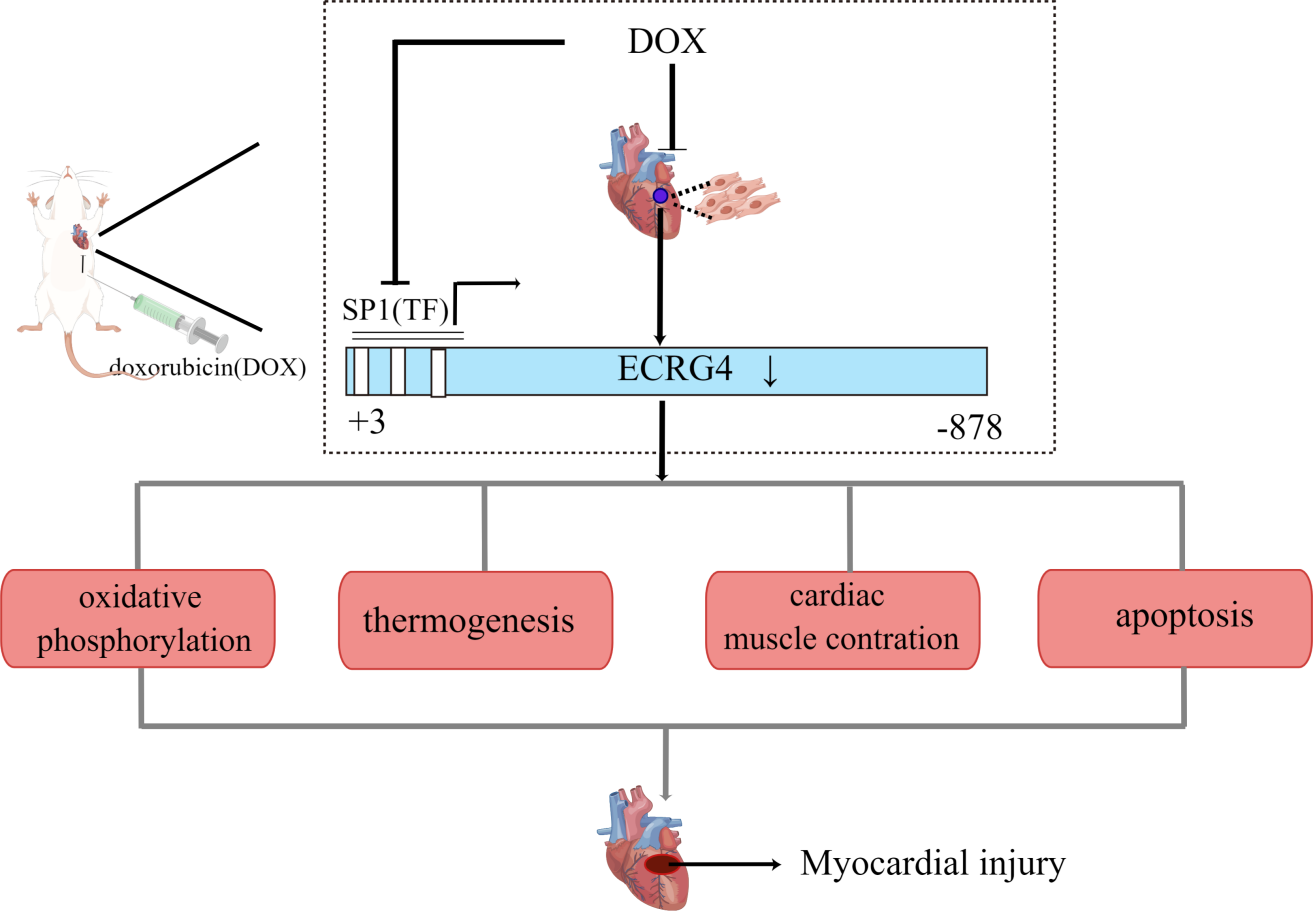
ECRG4 is involved in the mechanism of doxorubicin induced myocardial injury. Figure shows the 5’UTR gene sequence of rat ECRG4 -878/+3, which contains 3 potential SP1 transcription factors. Doxorubicin inhibited the expression of SP1 and reduced the binding of SP1 to the rat ECRG4 promoter, thereby decreasing the expression of ECRG4. Cardiomyocyte-specific loss of Ecrg4 significantly enriched the DEPs in the signaling pathways (oxidative phosphorylation, apoptosis, thermogenesis, and cardiac muscle contraction, among others) commonly involved in DOX-induced cardiotoxicity.

Additionally, certain drugs may affect ECRG4 expression and cause atrial fibrillation. Doxorubicin(DOX) is well-known for cardiotoxic effects, including atrial fibrillation. According to a prospective study by Kilickap and colleagues, DOX-containing regimens caused arrhythmia in 19 patients (65.5%) of 29 patients with various cancers, of whom 3 patients (10.3%) had paroxysmal AF (48). In a previous study, Long et al. investigated the role of ECRG4 in AF and myocardial injury induced by DOX (49). DOX decreased endogenous ECRG4 gene expression in the heart and cultured neonatal rat cardiomyocytes. Further, cardiomyocyte-specific conditional ECRG4 knockout mice showed increased sensitivity to DOX-induced cardiotoxicity due to abnormal signaling pathways, including Oxidative phosphorylation, Thermogenesis, Diabetic cardiomyopathy, Cardiac muscle contraction (**Figure 3**). This study suggests that ECRG4, which is constitutively expressed in the heart, can maintain cardiac homeostasis and protect cardiomyocytes from the cardiac toxicity caused by DOX (49). Taken together, the high incidence of AF in tumor patients may be related to ECRG4 expression. Therefore, it is crucial to investigate how ECRG4 maintains cardiac homeostasis and regulates cardiac rhythm.

Studies on ECRG4 in atrial fibrillation have also been conducted in animal models. Huang et al. previously found that the expression of ECRG4 was significantly decreased in a rapid atrial pacing-induced canine AF model, suggesting that ECRG4 participates in the pathogenesis of AF (44). In addition, another study found that 24 hours after rapid electrical stimulation, ECRG4 was significantly downregulated in mouse atrial myocytes HL1 (50). Furthermore, The International Mouse Phenotyping Consortium revealed that ECRG4 knockout mice have a shorter QRS complex duration, highlighting the role of ECRG4 in heart rate/rhythm regulation. Collectively, the above findings indicate that both tachyarrhythmias and rapid pacing can induce a significant decrease in the expression of ECRG4, and the decrease in ECRG4 further leads to atrial remodeling, which is essential for the generation and maintenance of atrial fibrillation.

## Atrial fibrillation and rhythm control regulated by ECRG4: Possible mechanisms

### ECRG4 affects multiple cardiac ion channels expression

ECRG4 knockdown in neonatal atrial myocytes significantly upregulated the expression of calcium-binding protein family gene (s100a1, s100a8), while downregulating gap channel protein-1(Gjal) expression, leading to a significant shortening of action potential duration (APD50 and APD90) (**Figure 2**) (30). Calcium homeostasis plays an important regulatory role in myocardial remodeling; s100a1 and sl00a8, members of the S100 calcium-binding protein family and expressed by cardiomyocytes, are key to regulating the Ca^2+^ concentration in cardiomyocytes (51). and in patients with atrial fibrillation, the shortened duration of the action potential can trigger and result in a continuous reentry loop. The shortening of action potential duration is mainly caused by an increase in K^+^ outward current and/or a decrease in inward current induced by Ca^2+^ during repolarization, and in patients with atrial fibrillation, the shortened duration of the action potential can trigger and result in a continuous reentry loop (52). In human-induced pluripotent stem cell-derived cardiac cells (hiPSC-CMs), ECRG4 knockdown using siRNA also altered the expression of multiple ion channels: SCN5A (sodium channel), KCNH2 (HERG channel) and KCND3 (transient outward K channel) were reduced, while KCNN4 (SK4 channel) and HCN2 (funny channel) were increased (53). Taken together, the above studies suggest that ECRG4 may be involved in atrial fibrillation *via* regulating these channels.

### ECRG4 affects the expression of matrix metalloproteinases

Studies have shown that Matrix metalloproteinase play an important role in the development of hypertension and atrial fibrillation by affecting the degradation of the extracellular matrix (54). MMP3 and MMP9 were increased in patients with recurrent AF within one year after electrical cardioversion (55). Studies have shown that MMP-9 is significantly higher in obese patients with paroxysmal atrial fibrillation than in obese patients alone. With the increase of MMP-9 in obese patients exceeding 285ng/ml, the occurrence of AF can be predicted with a sensitivity of 74.5% and specificity of 94% (56). It has been shown that ECRG4 can regulate the expression of MMPs in various organs and tissues. In oral squamous cell carcinoma, ECRG4 down-regulated the expression of matrix metalloproteinases (MMP-9 and MMP-13) (57). In atrial myocytes, ECRG4 knockdown significantly upregulated the expression of matrix metalloproteinase3 (MMP3) (30), which may contribute to atrial fibrillation.

### ECRG4 and immune inflammatory reactions

A substantial amount of evidence suggests that the onset and progression of AF are strongly linked to inflammation (58–61). Epidemiological studies have shown that compared with subjects with normal sinus rhythm, c-reactive protein and inflammatory cytokines such as TNF-α, IL-1β, IL-8, IL-6 and monocyte chemoattractant protein-1 (MCP-1) were significantly upregulated in patients with atrial fibrillation (59). Inflammatory mediators can disrupt cellular calcium homeostasis, activate and promote fibrosis, inhibit gap junction protein (Gjal)-mediated cell-cell communication (GJIC), and induce cardiomyocyte necrosis and apoptosis (58, 62, 63). ECRG4 knockdown in atrial myocytes significantly increased the production of pro-inflammatory genes. In recent years, a large amount of literature supports the involvement of ECRG4 in inflammation, injury, and infection. ECRG4 is a candidate chemokine that is highly expressed on leukocytes and regulates early neutrophil recruitment and subsequent CD44-mediated inflammatory decline, making ECRG4 a therapeutic target for inflammatory diseases (64). ECRG4 is also expressed on the cell surface of epithelial cells (12, 13, 65, 66). Cell surface ECRG4 is expressed in quiescent tissue and may have a “sentinel” function to monitor homeostasis, measure pro-inflammatory responses to injury and infection, and thus remain quiescent (11–13, 65). When infection or inflammation occurs, the protease activates and initiates the downregulation of ECRG4 gene expression, which is released from the cell surface in a processed form (9, 11, 67). Other studies have shown that ECRG4 has a physiological role in measuring parenchymal and inflammatory responses to traumatic brain injury, with small needle wounds leading to a temporary reduction and full recovery of ECRG4 within 24-48 hours (13, 65). These findings suggest that low expression of ECRG4 may contribute to the development of atrial fibrillation through an inflammatory response.

### ECRG4: Potential clinical target in tumor and arrhythmia

ECRG4 not only has a tumor suppressor effect but also cytokine-like functions. ECGG4 is found in a variety of bodily fluids, including blood, urine, saliva, pleural effusion, and cerebrospinal fluid. Decreased concentrations of ECRG4 in body fluids may indicate cancer development, suggesting that ECRG4 may be a biomarker for predicting cancer occurrence. Previous studies have found that methylated ECRG4 cDNA has promising diagnostic and clinical translational potential (68, 69). Meanwhile, promoter methylation determines ECRG4 expression status, and its aberration could help detect early cancer and predict severity (70). These findings show that ECRG4 can be used as a biomarker for cancer diagnosis as well as predicting staging and invasiveness. The wide distribution and diverse functions of ECRG4 make it an ideal target for drug therapy. ECRG4 has several functions, including tumor suppression, heart rhythm regulation, cardiac homeostasis maintenance, and involvement in the aging process. Overexpression of ECRG4 has been found to increase the sensitivity of gastric cancer cell line, SGC-7901 to 5-FU and NPC cell line, CNE1 to cisplatin, thereby improving the therapeutic effect of chemotherapy drugs (71, 72). Upregulation of ECRG4 expression or activity may also be used to treat diseases characterized by tissue dysfunction caused by attenuated ECRG4 expression. Since down-regulation of ECRG4 is associated with atrial fibrillation, up-regulating ECRG4 expression may aid in treating atrial fibrillation. Moreover, inhibition of ECRG4 can counteract senescence-associated cellular senescence. Interestingly, ECRG4 expression is generally silenced by promoter methylation, which demethylating agents can reactivate. There are currently two types of demethylation drugs, nucleoside DNMT inhibitors and non-nucleoside DNMT inhibitors, the efficacy of which is still being investigated. Furthermore, ECRG4 is a secretory protein that attaches to the cell surface and undergoes proteolysis to achieve biological activation. Therefore, high-affinity receptor agonists or protease inhibitors of ECRG4 are attractive targets for future drug development.

## Perspectives

ECRG4 was initially known for its antitumor function, but as research progressed, its role in various physiology and pathology was gradually revealed, as was its role in the heart. The literature on atrial fibrillation induced by down-regulation of ECRG4 was summarized, and it was found that down-regulation of ECRG4 could induce atrial fibrillation through affecting ion channels, MMPs expression, and activating inflammatory response. ECRG4 is involved in the mechanism of doxorubicin-induced myocardial injury, which suggests that ECRG4 has myocardial protective function. In conclusion, ECRG4 can regulate rhythm, maintain cardiac homeostasis and protect myocardium. This study comprehensively reviewed the biological background of ECRG4 gene and its expression in cardiovascular system, focusing on the possible mechanism of ECRG4’s involvement in the formation of atrial fibrillation, which provides a new idea for reducing perioperative atrial fibrillation and myocardial injury in patients with esophageal cancer, gastric cancer and other tumors.

The uniqueness of ECRG4 makes it a potential target for precision medicine. Current research is primarily based on *in vitro* experiments, such as studies in KO or transgenic mouse models, which are extremely useful in determining the role of ECRG4 *in vivo*. The transition from basic research to clinical application necessitates a lengthy process of clarification and validation. Future research will need to decipher the mechanisms of action of ECRG4 and its signaling pathways. Continued development of new targeted drugs is expected to benefit not only the treatment of esophageal cancer, gastric cancer, and other cancers but also the treatment of cardiovascular diseases such as atrial fibrillation and myocardial injury.

## Author contributions

ZZ, WW, and YZ contributed equally to this work. All authors contributed to the article and approved the submitted version.
